# Hearing loss associated with CDC42 in mice and humans (Takenouchi–Kosaki syndrome): CDC42 and RHOQ synergistically function in cochlear hair cells

**DOI:** 10.1016/j.jbc.2026.113402

**Published:** 2026-08-07

**Authors:** Takehiko Ueyama, Akira Ganaha, Shunkou Kurasawa, Hiroaki Mohri, Takashi Nakamura, Shigefumi Morioka, Fumiya Kawama, Shota Kitayama, Naoki Oishi, Tadashi Kaname, Keiji Tabuchi, Kenjiro Kosaki, Toshiki Takenouchi, Hirofumi Sakaguchi

**Affiliations:** 1 Laboratory of Molecular Pharmacology, Biosignal Research Center, Kobe University, Kobe, Japan; 2 Department of Otolaryngology-Head and Neck Surgery, International University of Health and Welfare Narita Hospital, Narita, Japan; 3 Department of Otorhinolaryngology-Head and Neck Surgery, University of the Ryukyus, Inowan, Japan; 4 Department of Otorhinolaryngology, Head and Neck Surgery, Keio University School of Medicine, Tokyo, Japan; 5 Department of Genome Medicine, National Center for Child Health and Development, Tokyo, Japan; 6 Department of Otolaryngology-Head and Neck Surgery, University of Tsukuba, Tsukuba, Japan; 7 Center for Medical Genetics, Keio University School of Medicine, Tokyo, Japan; 8 Department of Pediatric Neurology, Okayama University Graduate School of Medicine, Dentistry and Pharmaceutical Sciences, Okayama, Japan

**Keywords:** actin, apical junctional complex, CDC42, cochlear hair cell, cofilin, RHOQ, sensorineural hearing loss, Takenouchi–Kosaki syndrome, tight junction, stereocilia, Rho-family small GTPase

## Abstract

CDC42 is involved in multiple signaling pathways, including actin organization and polarity. We previously reported progressive sensorineural hearing loss (SNHL) in inner ear hair cell (HC)-specific *Cdc42*-knockout (*Atoh1*-*Cre*^+/−^;*Cdc42*^*flox*/*flox*^) mice. However, the phenotype was milder than expected, suggesting possible redundancy with other Rho-family GTPases. Thereafter, Takenouchi–Kosaki syndrome (TKS), caused by *de novo CDC42* mutations and manifesting as SNHL, was reported, in which the p.Y64C mutation was speculated to be constitutively active. However, the relationship between CDC42 status and hearing phenotypes in TKS remains unclear. Using cell models, mouse models, and patient data, we propose that impaired and/or dysregulated cycling between GDP/inactive and GTP/active forms, through either loss-of-function or constitutive activation, can lead to SNHL. Furthermore, to test redundancy, we generated HC-specific *Cdc42*;*RhoQ* double-knockout (*Atoh1*-*Cre*^+/−^;*Cdc42*^*flox*/*flox*^;*RhoQ*^*flox*/*flox*^) mice, which revealed synergistic roles of CDC42 and RHOQ in cochlear HCs. Supporting this synergy, MDCK cells with CDC42 and RHOQ double knockdown showed greater phospho-cofilin, a key regulator of actin turnover, elevation than single knockdowns.

Cell division cycle 42 (CDC42) is a member of the Rho family of small GTPases and is involved in multiple signaling pathways, including cytoskeleton organization and establishment of cell polarity at cellular and tissue levels (1, 2). Its function depends on tightly regulated cycling between GDP-bound inactive and GTP-bound active states, occurring in precise spatiotemporal contexts (1, 2), which is essential for the development and maintenance of actin-based structures. This regulation is controlled by three factors: guanine nucleotide exchange factors (GEFs), GTPase-activating proteins (GAPs), and guanine nucleotide dissociation inhibitors (GDIs) (2, 3). Recently, we showed that CDC42 is required for cochlear hair cell (HC) maintenance in inner ear HC-specific *Cdc42*-knockout mice (*Cdc42*-KO, *Atoh1*-*Cre*^+/−^;*Cdc42*^*flox*/*flox*^) (4). Although morphological development was normal at postnatal day 8 (P8), *Cdc42*-KO mice developed progressive sensorineural hearing loss (SNHL) because of HC loss accompanied by abnormalities in stereocilia and apical junctional complexes (AJCs) (4). We also found that active CDC42 is localized to stereocilia and AJCs in cochlear HCs (4). However, the hearing phenotype was milder than expected, suggesting functional redundancy with other CDC42-related GTPases, including Ras homolog family member Q (RHOQ, also referred to as TC10) and/or Ras homolog family member J (RHOJ), which belong to the same subgroup of Rho-family small GTPases as CDC42, although they were not investigated. In a cell-type-specific RNA-sequence study (5), *RhoQ* mRNA was almost equally expressed in HCs and non-HCs in the developing inner ear (embryonic day 16 [E16]–P7); in sharp contrast, *RhoJ* mRNA was almost exclusively expressed in non-HCs. However, *RhoQ* mRNA expression levels in cochlear HCs are approximately one-quarter to one-fifth of those of *Cdc42* mRNA (5), making its compensatory contribution uncertain.

After the report of HC-specific *Cdc42*-KO mice (4), Takenouchi–Kosaki syndrome (TKS), which is caused by *de novo* mutations in *CDC42* and characterized by various brain anomalies, dysmorphic craniofacial features, hematological anomalies including macrothrombocytopenia and immunological dysfunction with recurrent infection, hypothyroidism, and SNHL, was reported. The first two patients carried the p.Y64C mutation (6, 7). Subsequently, Martinelli *et al.* reported 15 patients with nine different *CDC42* mutations and classified TKS into three groups according to the mutants’ structure and function (8). Group I (original/typical TKS) mutants (Y64C, R66G, and R68Q) are located in the switch II region of CDC42 (amino acid [aa] 62 to 68 (9, 10)), which mediates CDC42 binding to effectors and regulators. Group II mutants (C81F/Y, S83P, and A159V) lie within or near the nucleotide-binding pocket. Finally, Group III mutants (I21T, Y23C, and E171K) affect interactions with effectors containing CDC42/RAC-interacting binding (CRIB) motif (8). Two alternatively spliced CDC42 isoforms with distinct C-terminal regions (aa 163–191; a marked difference is ^182^PKKSRRCVLL^191^X in isoform 1 and ^182^TQPKRKCCIF^191^X in isoform 2) (11), exist in humans and mice; isoform 1 (exons 1–5 and 7) is ubiquitously expressed, while isoform 2 (exons 1–6) is predominantly expressed in the brain (12, 13, 14). Mutations affecting only the C-terminus of isoform 1 spare the intact brain-dominant isoform 2, leading to weakened actinopathies, including no SNHL (15). Indeed, nine patients with *CDC42* mutations in the C-terminus of isoform 1 (R186C, C188Y, and X192CfsX24) showed clinical features distinct from typical TKS (13, 15, 16). This condition is characterized by neonatal onset of pancytopenia, autoinflammation, rash, and hemophagocytic lymphohistiocytosis (HLH) (NOCARH syndrome). Since actin-based structures undergo continuous turnover regulated by CDC42 activity, mutants with impaired GDP/GTP regulation, such as constitutively active and loss-of-function variants, are thought to underlie CDC42-related disorders (1, 4, 8). Although the Y64C mutant has been suggested to be constitutively active (17), the functional status of CDC42 mutants in TKS, especially their effects on the hearing phenotype, remains unclear.

In the present study, we examined Group I CDC42 mutants associated with classical TKS and investigated the relationship between CDC42 status and SNHL in a new patient with p.R68Q as well as reevaluation of Japanese patients carrying p.Y64C. We propose that CDC42 with impaired conversion between the GDP/inactive and GTP/active forms, including loss-of-function (*i*.*e*., *Cdc42*-KO) and constitutively active mutants (*i*.*e*. Y64C and R68Q), likely causes SNHL in TKS. Moreover, we generated *Atoh1*-*Cre*^+/−^;*Cdc42*^*flox*/*flox*^;*RhoQ*^*flox*/*flox*^ (HC-specific *Cdc42*;*RhoQ*-double KO [DKO]) mice to examine functional redundancy in hearing and demonstrated that CDC42 and RHOQ function synergistically in cochlear HCs.

## Results

### A patient with TKS possessing CDC42(R68Q) mutation

A 2-year-old Japanese female was referred to our hospital for hearing loss (HL). The mother’s pregnancy and delivery at 38 weeks of gestation were uneventful, and no family history of HL was reported. Profound bilateral HL was confirmed based on auditory brainstem response (ABR) at 3 months of age (Fig. S1*A*) and pure-tone audiometry at 4 years of age (Fig. S1*B*). High-resolution temporal bone computed tomography revealed no inner or middle ear anomalies (Fig. S1*C*). The patient had been using hearing aids in both ears since 1.5 years of age. Examination revealed dysmorphic facial features, intellectual disability, camptodactyly, cardiac anomalies (double-outlet right ventricle), and congenital hypothyroidism. Whole-exome sequencing demonstrated a *CDC42* variant (NM_001791, exon4:c.G203A: p.R68Q), which was present in the patient but not in the parents and was confirmed by direct sequence analysis (Fig. S1*D*). The patient was diagnosed with TKS based on the clinical and genetic findings. No family history of TKS was observed. May–Grünwald-Giemsa-stained blood smears confirmed macrothrombocytopenia. The patient had a history of recurrent acute otitis media in both ears. Patient information (Patient 4) and patients with TKS reported in Japan (re-evaluated in the present study, Patients 1–3) are summarized in Table 1.

**Table 1:** Summary of patients with TKS reported in Japan, including the patient with p.R68Q mutation in this study

Clinical features	Patient 1 Takenouchi *et al.* [6]	Patient 2 Takenouchi *et al.* [7]	Patient 3 Motokawa *et al.* [72]	Patient 4 presented patient
Genotype	p.Y64C	p.Y64C	p.Y64C	p.R68Q
Age/Sex	21/Female	22/Female	18/Female	10/Female
Dysmorphic facial features	+	+	+	+
Brain anomaly	+	+	+	−
Macrothrombocytopenia	+	+	+	+
Hypothyroidism	−	−	+	+
Camptodactyly	+	+	+	+
Heart anomaly	−	Patent ductus arteriosus	−	Double outlet right ventricle
Hearing				
Sensorineural hearing loss	+	+	+	+
ABR response threshold^a^	70	No response	80	No response
Hearing level^b^	severe	profound	severe	profound
CT/MRI findings of middle/inner ear and IAC^c^	normal	normal	Bilateral cochlear nerve hypoplasia, Stenosis of IAC	normal

a Auditory brainstem response (ABR) threshold were visually inspected by wave V.

b Hearing level was described as follows: normal, ≤25 dB; mild impairment, 26–40 dB; moderate impairment, 41 to 60 dB; severe impairment, 61 to 80 dB; and profound impairment, ≥81 dB.

c IAC, Internal auditory canal.

### Phenotypic properties of CDC42 mutants in cells

**Figure 1:**
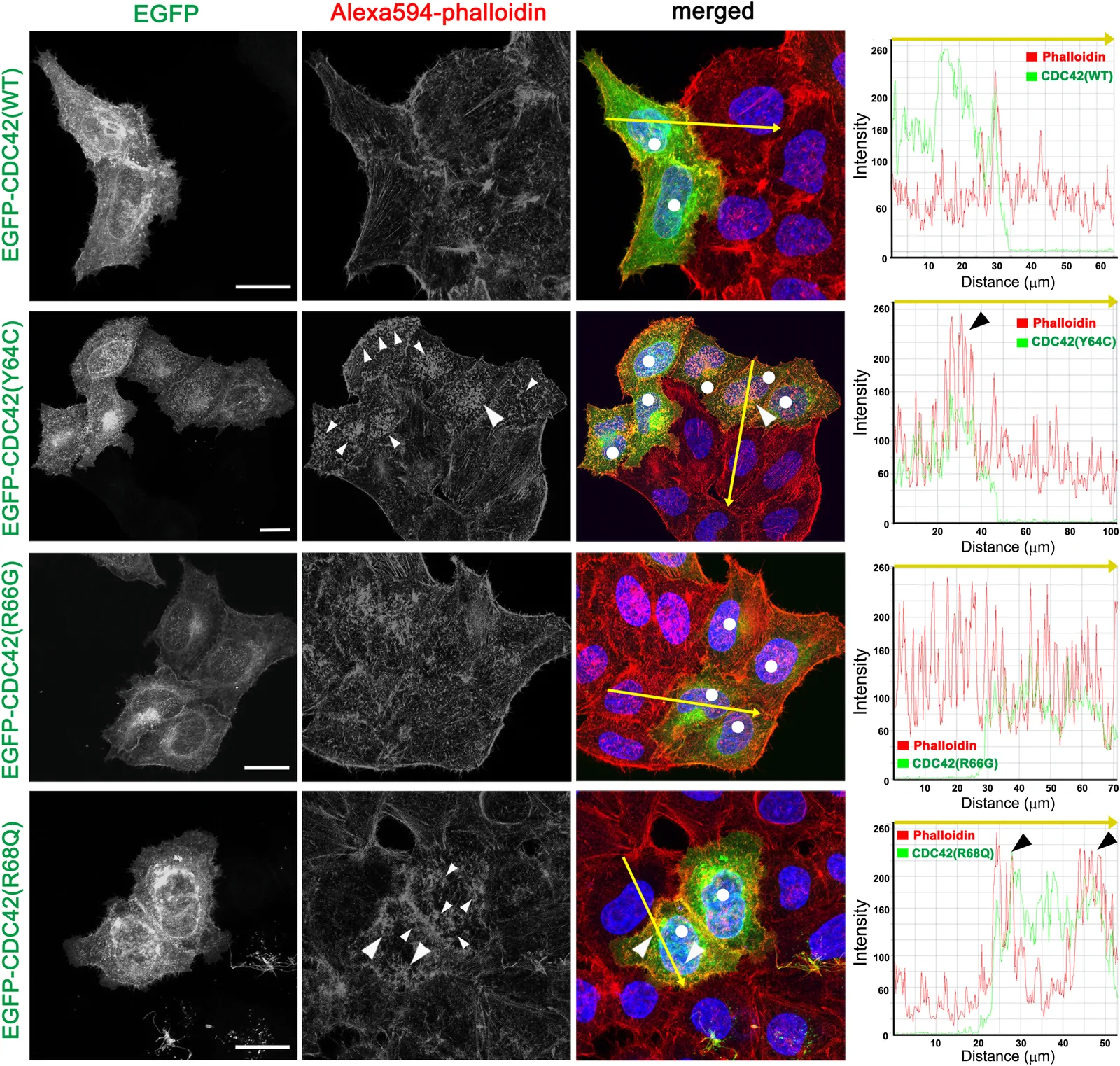
**Cellular phenotypes induced by overexpression of EGFP-CDC42 variants in HeLa cells.** HeLa cells were transfected with EGFP-CDC42(WT), EGFP-CDC42(Y64C), EGFP-CDC42(R66G), or EGFP-CDC42(R68Q). Forty-eight hours later, the cells were fixed, stained with Alexa594-conjugated phalloidin and DAPI. Orthogonal (z-stacked) images of each EGFP-tagged CDC42 protein are shown. Cells transfected with EGFP-CDC42(Y64C) or EGFP-CDC42(R68Q) show enhanced phalloidin staining (microvilli indicated by arrowheads) compared with surrounding non-transfected cells. Circles: cells expressing EGFP-tagged protein. The fluorescence intensity profiles across the *yellow arrow* for *green* (EGFP) and *red* (phalloidin) channels were analyzed using Zen software and are shown in the graphs (*far right*). The x-axis represents the distance from the starting point of the *yellow arrows* in the images to their tips. *Arrowheads* in the graphs for EGFP-CDC42(Y64C) and EGFP-CDC42(R68Q) indicate the positions of microvilli. Scale bars, 20 μm. Three-dimensional movies are available as supplementary materials (Movies S1–S4). Abbreviations: CDC42, cell division cycle 42; DAPI, 4′,6-diamidino-2-phenylindole; EGFP, enhanced *green* fluorescent protein; WT, wild type.

As an initial step, we examined the activity status of Group I TKS-associated CDC42 mutants—CDC42(Y64C), CDC42(R66G), and CDC42(R68Q)—in comparison with CDC42(WT) in HeLa cells, which served as a model system for investigating actin cytoskeletal structures, such as microvilli and stress fibers (18, 19, 20). Stereocilia are an exquisitely organized subtype, with a tightly regulated length and shape, of microvilli and filopodia (19, 21), and we previously reported CDC42-dependent developmental and morphological regulation of microvilli and stereocilia (4). Although cells expressing EGFP-CDC42(WT) or EGFP-CDC42(R66G) showed no enhanced staining of phalloidin compared with the surrounding non-transfected cells (Fig. 1, Movies S1–S4), those expressing EGFP-CDC42(Y64C) and EGFP-CDC42(R68Q) showed markedly enhanced dot-like phalloidin staining, indicating the presence of microvilli on the apical surface (Fig. 1, Movies S2 and S4). To determine whether cells expressing EGFP-CDC42 mutants exhibited actin accumulation compared with the surrounding non-expressing cells, the fluorescence intensity profiles across the yellow arrow for green (EGFP) and red (phalloidin) channels were analyzed using Zen software and plotted (Fig. 1, far right). Cells expressing CDC42(Y64C) or EGFP-CDC42(R68Q) showed higher phalloidin fluorescence intensity than the surrounding EGFP-negative cells, and EGFP and phalloidin signals colocalized at microvilli. In contrast, cells expressing EGFP-CDC42(WT) or EGFP-CDC42(R66G) did not show an apparent increase in phalloidin intensity compared with the surrounding EGFP-negative cells. Supporting these observations, a pulldown assay detecting GTP-bound (active) CDC42 showed that EGFP-tagged CDC42(Y64C), CDC42(R66G), and CDC42(R68Q) retained the active conformation more than EGFP-CDC42(WT) (Fig. S2). These results suggested that at least CDC42(Y64C) and CDC42(R68Q) are active mutants for microvilli.

### Marked rescue of the tight junction phenotype in MDCK^Cdc42KD^ cells by CDC42(Y64C) and CDC42(R68Q)

**Figure 2:**
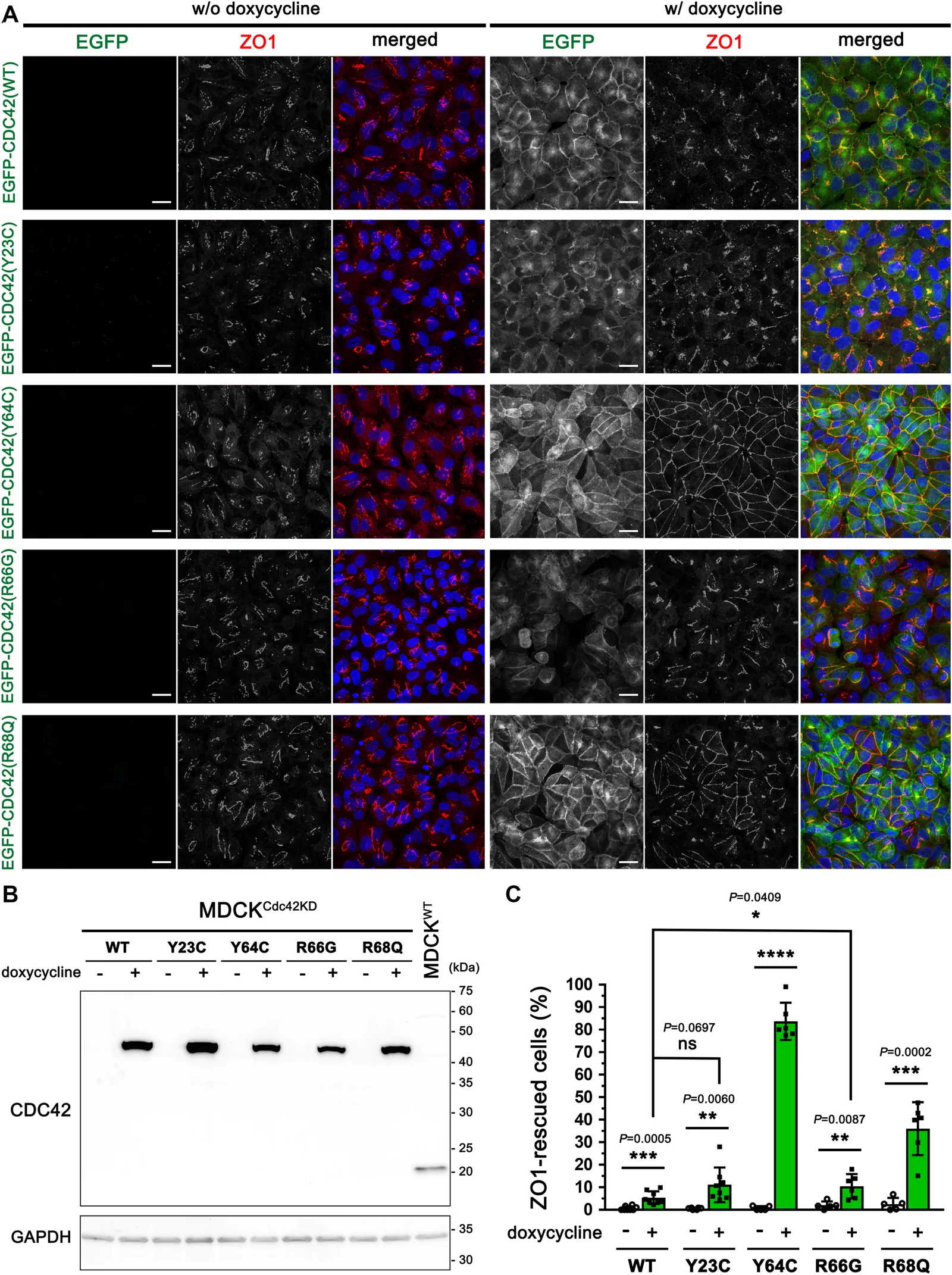
**CDC42(R68Q) and CDC42(Y64C) markedly rescue the TJ phenotype in MDCK^Cdc42KD^ cells.***A*, MDCK^Cdc42KD^ cells stably expressing tetEGFP-CDC42(WT), tetEGFP-CDC42(Y23C), tetEGFP-CDC42(Y64C), tetEGFP-CDC42(R66G), or tetEGFP-CDC42(R68Q) were cultured on 35-mm glass-bottom dishes and incubated for 48 h with doxycycline (100 ng/ml) to induce transgene expression. The cells were then fixed and immunostained with an anti-ZO-1 antibody, followed by DAPI counterstaining, and imaged using confocal microscopy. Scale bars, 20 μm. *B*, Stable expression of tetEGFP-CDC42(WT), tetEGFP-CDC42(Y23C), tetEGFP-CDC42(Y64C), tetEGFP-CDC42(R66G), or tetEGFP-CDC42(R68Q) in MDCK^Cdc42-KD^ cells were confirmed by immunoblotting with an anti-Cdc42 antibody. GAPDH was used as a loading control. *C*, quantification of cells with rescued ZO-1 localization. Significant difference was detected between groups without and with doxycycline induction in all CDC42 variants (Student’s *t* test). After doxycycline induction, a significant difference was observed between CDC42(WT) and CDC42(R66G) cells, but not between CDC42(WT) and CDC42(Y23C) cells. The symbols in the graph indicate the number of experiments performed (n >5). *p* values by Student’s *t* test are shown in the graph. Abbreviations: CDC42, cell division cycle 42; DAPI, 4′,6-diamidino-2-phenylindole; EGFP, enhanced green fluorescent protein; KD, knockdown; ns, not significant; TJ, tight junction; ZO-1, zonula occludens-1.

The CDC42 GEF Tuba localizes to tight junctions (TJs) through its interaction with ZO-1, and it stimulates CDC42-Wiskott–Aldrich Syndrome protein (WASP)-driven actin polymerization (22, 23). We previously reported that MDCK cells with stable *Cdc42* knockdown (MDCK^Cdc42KD^) show disrupted ZO-1 staining (4). To further examine CDC42 mutant function, we established MDCK^Cdc42KD^ cells stably expressing EGFP-tagged CDC42(WT), CDC42(Y23C), Cdc42(Y64C), CDC42(R66G), or CDC42(R68Q) following doxycycline induction (Fig. 2, *A* and *B*). Expression of all CDC42 variants significantly affected ZO-1 staining, and CDC42(Y64C)- or CDC42(R68Q)-expressing cells showed strong rescue of ZO-1 localization compared with uninduced cells (Fig. 2, *A* and *C*). The difference between WT and CDC42(Y23C) showed a trend (*p* = 0.0697), whereas the difference between WT and CDC42(R66G) was significant (*p* = 0.0409; Fig. 2, *A* and *C*). These results suggest that CDC42(Y64C) and CDC42(R68Q) are strongly active mutants, while CDC42(Y23C) and CDC42(R66G) are weaker active mutants relative to CDC42(WT).

### Additional RhoQ deletion exacerbates hearing phenotypes in Cdc42-KO mice

**Figure 3:**
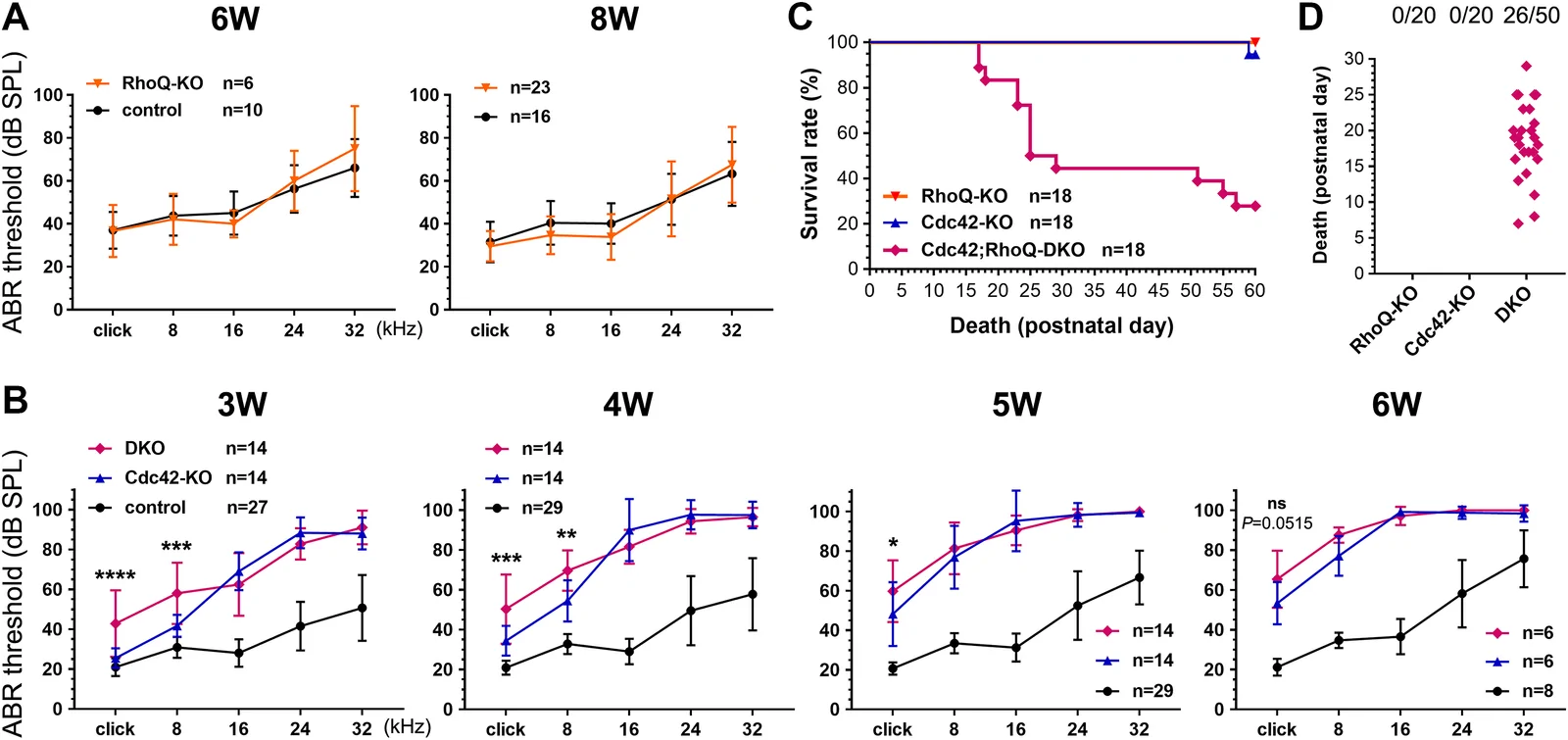
***RhoQ* deletion exacerbates the hearing phenotype of *Cdc42*-KO mice.***A*, ABR thresholds do not differ significantly between WT and *RhoQ*-KO mice at 6 and 8 weeks of age (6W and 8W). Two-way ANOVA with Bonferroni’s *post hoc* test. The number of evaluated mice is shown in the graphs. *B*, ABR thresholds of WT, *Cdc42*-KO, and *Cdc42*;*RhoQ*-DKO mice evaluated at weeks 3, 4, 5, and 6W showing significant differences between *Cdc42*-KO and *Cdc42*;*RhoQ*-DKO mice. Two-way ANOVA with Tukey’s *post hoc* test (∗∗∗∗*p* < 0.0001 and ∗∗∗*p =* 0.0002 at 3W; ∗∗∗*p =* 0.0006 and ∗∗*p =* 0.0011 at 4W; ∗*p =* 0.0149 at 5W). The number of evaluated mice is shown in the graphs. *C*, survival rates and ages at death of *Cdc42*-KO, *RhoQ*-KO, and *Cdc42*;*RhoQ*-DKO mice until postnatal day 60 (P60; n = 18). One Cdc42-KO mouse died, whereas no RhoQ-KO mice died during this period. *D*, distribution of age at death for *Cdc42*-KO (n = 20), *RhoQ*-KO (n = 20), and *Cdc42*;*RhoQ*-DKO (n = 50) mice until P30. A total of 26 of 50 *Cdc42*;*RhoQ*-DKO mice died by P30. Abbreviations: ABR, auditory brainstem response; DKO, double knockout; KO, knockout; ns, not significant; P, postnatal day; W, weeks; WT, wild type.

We next examined functional redundancy between CDC42 and RHOQ. Reanalysis of our previous DNA microarray data from the whole cochlea of WT mice showed *RhoQ*/*Cdc42* expression ratios of 16847.4/64534.1 (1/3.83) at P6 and 5869.2/6740.6 (1/1.15) at P15 (24). In contrast, cell type-specific RNA-sequencing analysis by Scheffer *et al.* (5) showed expression ratios of approximately 1/4 to 5 at E16–P7 in cochlear HCs, with mRNA expression of *RhoQ* and *Cdc42* peaking at P0 and P4, respectively. ABR evaluation revealed normal hearing function in *RhoQ*-KO mice at 6 and 8 weeks of age compared with control mice (Fig. 3*A*). In contrast, *Cdc42*;*RhoQ*-DKO mice (*Atoh1*-*Cre*^+/−^;*Cdc42*^*flox*/*flox*^;*RhoQ*^*flox*/*flox*^) showed more severe hearing impairment compared with *Cdc42*-KO mice at 3 weeks of age, especially at low frequencies (click and 8 kHz) (Fig. 3*B*). The significance was highest at 3 weeks of age, gradually decreased, and was absent at 6 weeks (*p* = 0.0515 at click) (Fig. 3*B*). To explore the cause of this phenomenon (*i*.*e*., the significant difference observed at 3 weeks but not at 6 weeks), we examined mouse survival. By P60, 12 of 18 *Cdc42*;*RhoQ*-DKO mice had died, whereas only one *Cdc42*-KO mouse and no *RhoQ*-KO mice had died (Fig. 3*C*). Because deaths were clustered during two periods (P15–P30 and P50–P60, Fig. 3*C*), we performed a more detailed survival analysis up to P30. By P30, 26 of 50 *Cdc42*;*RhoQ*-DKO mice had died, whereas no deaths were observed in either the *Cdc42*-KO or *RhoQ*-KO groups (Fig. 3*D*). These results suggest that *Cdc42*;*RhoQ*-DKO mice with more severe phenotypes may die earlier than those with milder phenotypes, thereby reducing the apparent hearing differences at later ages because of survivor bias.

### Deletion of RhoQ in addition to Cdc42 results in the appearance of a fourth row of outer HCs in Cdc42;RhoQ-DKO mice

**Figure 4:**
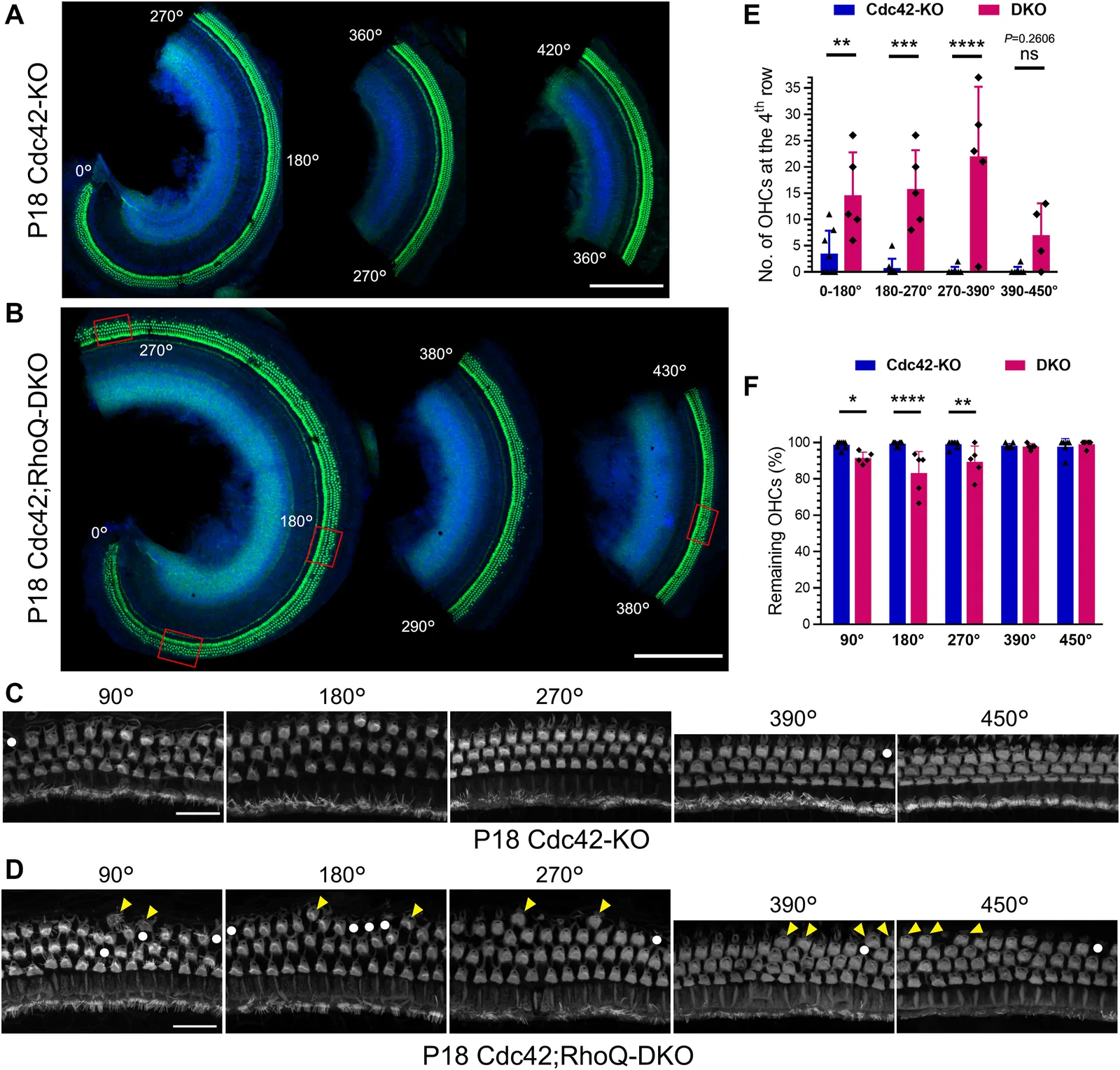
***RhoQ* deletion exacerbates PCP abnormalitiesand OHC lossin *Cdc42*-KO mice.** Whole-mount cochleae (decalcified) were prepared from P18 *Cdc42*-KO (*A*, *C*) and *Cdc42*;*RhoQ*-DKO (*B*, *D*) mice and stained with Alexa488-conjugated phalloidin with DAPI. Representative magnified images are shown in *C* and *D*. For *Cdc42*;*RhoQ*-DKO mice, magnified images were obtained from the same sample shown in *B*, with the locations indicated by *rectangles*. Scale bars, 250 μm in *A* and *B*; 20 μm in *C* and *D*. The number of OHCs in the fourth row (*E*; ∗∗*p =* 0.0069, ∗∗∗*p =* 0.0002, and ∗∗∗∗*p* < 0.0001) and the percentage of remaining OHCs (*F*; ∗*p =* 0.0395, ∗∗∗∗*p* < 0.0001, and ∗∗*p =* 0.0025) are plotted. Significant differences were detected using one-way ANOVA with Bonferroni’s *post hoc* test. The symbols in the graph indicate the number of experiments performed (n >4). Abbreviations: DKO, double knockout; KO, knockout; ns, not significant; OHC, outer hair cell; P, postnatal day; PCP, planar cell polarity.

We previously reported outer hair cell (OHC) loss at 4 weeks of age in *Cdc42*-KO mice (4). However, we detected no apparent planar cell polarity (PCP) impairment, consistent with another study (25). In the present study, we compared OHC loss and PCP between *Cdc42*-KO and *Cdc42*;*RhoQ*-DKO mice at P18, when cochlear HCs and the organ of Corti development are complete (26, 27, 28, 29, 30). In WT mice, OHCs lie in three rows (30, 31). However, compared with *Cdc42*-KO mice, *Cdc42*;*RhoQ*-DKO mice showed a significant increase in fourth-row OHCs in the 0 to 180°, 180 to 270°, and 270 to 390° regions (Fig. 4, *A*–*E*). We observed no apparent hair bundle disorientation in cochlear HCs in *Cdc42*-KO or *Cdc42*;*RhoQ*-DKO mice (Fig. 4, *C* and *D*). Additionally, *Cdc42*;*RhoQ*-DKO mice showed significantly fewer remaining OHCs at 90°, 180°, and 270°, which correspond to low-frequency sound detection (<16 kHz), compared with *Cdc42*-KO mice (Fig. 4, *C*, *D*, and *F*).

### No significant rescue of the TJ phenotype in MDCK^Cdc42KD^ cells by RHOQs

**Figure 5:**
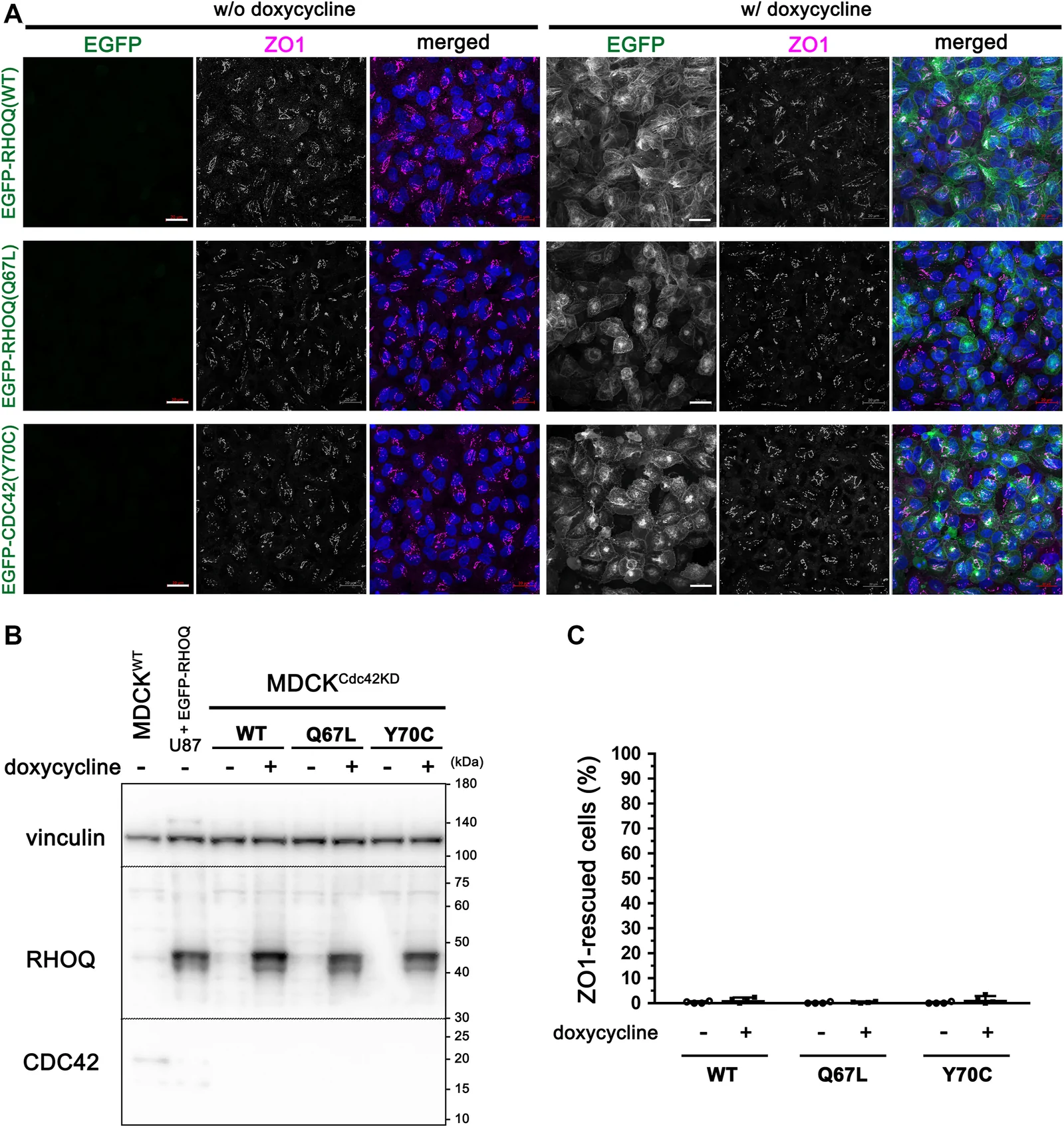
**RHOQ(WT), RHOQ(Q67L), or RHOQ(Y70C) do not rescue the TJ phenotype in MDCK^Cdc42KD^ cells.***A*, MDCK^Cdc42KD^ cells stably expressing EGFP-RHOQ(WT), EGFP-RHOQ(Q67L), or EGFP-RHOQ(Y70C) were cultured on 35-mm glass-bottom dishes and incubated for 48 h with doxycycline (100 ng/ml). The cells were then fixed and immunostained with an anti-ZO-1 antibody, followed by DAPI counterstaining, and imaged using confocal microscopy. Scale bars, 20 μm. *B*, stable expression of EGFP-RHOQ(WT), EGFP-RHOQ(Q67L), and EGFP-RHOQ(Y70C) in MDCK^Cdc42KD^ cells was confirmed by immunoblotting using an anti-RhoQ antibody. Vinculin was used as a loading control. *C*, quantification of the percentages of cells with rescued ZO-1 localization. No significant differences were detected between groups (Student’s *t* test, n = 4). Abbreviations: DAPI, 4′,6-diamidino-2-phenylindole; EGFP, enhanced green fluorescent protein; KD, knockdown; RHOQ, Ras homolog family member Q; TJ, tight junction; ZO-1, zonula occludens-1.

To examine CDC42–RHOQ synergy, we established MDCK^Cdc42KD^ cells stably expressing EGFP-tagged RHOQ(WT), RHOQ(Q67L) which is a constitutively active mutant (10), or RHOQ(Y70C) which is the corresponding mutant to CDC42(Y64C), following doxycycline induction (Fig. 5*A*). Expression was confirmed by immunoblotting using an anti-RHOQ antibody (Fig. 5*B*). None of the RHOQ constructs significantly restored ZO-1 staining (Fig. 5, *A* and *C*). However, transient KD of *RhoQ* by two different *RhoQ* siRNAs significantly impaired ZO-1 staining compared with controls (Fig. S3). These results suggest that RHOQ contributes to TJ formation but requires cooperation with CDC42.

### CDC42-KD and RHOQ-KD synergistically enhance phospho-cofilin levels in MDCK cells

**Figure 6:**
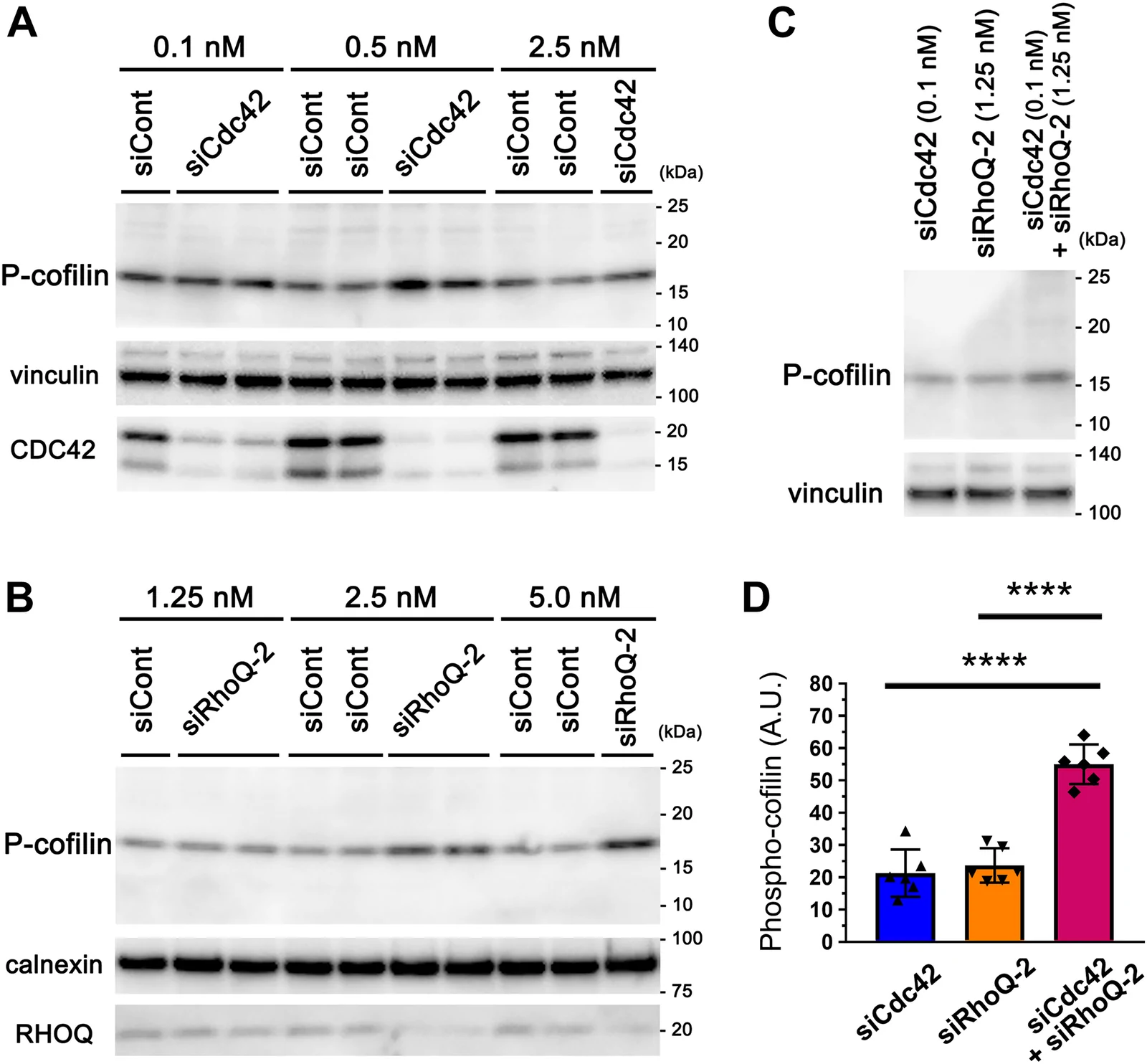
**CDC42-KD and RHOQ-KD synergistically enhance phospho-cofilin levels in MDCK cells.** WT MDCK cells were transfected with control or *Cdc42* siRNA (0.1, 0.5, or 2.5 nM) (*A*), control or *RhoQ-2* siRNA (1.25, 2.5, or 5.0 nM) (*B*), and *Cdc42* siRNA (0.1 nM), *RhoQ-2* siRNA (1.25 nM), or combination siRNAs of *Cdc42* (0.1 nM) and *RhoQ-2* (1.25 nM) (*C*). Forty-eight hours later, cell lysates were collected and subjected to immunoblotting for P-cofilin. KD of CDC42 and RHOQ was confirmed by immunoblotting using each antibody. Vinculin and calnexin were used as loading controls. *D*, Synergistic effects of double KD of CDC42 and RHOQ on P-cofilin levels were quantified (∗∗∗∗*p* < 0.0001, one-way ANOVA with Tukey’s *post hoc* test [n = 6]). Abbreviations: CDC42, cell division cycle 42; KD, knockdown; MDCK, Madin–Darby canine kidney; p-cofilin, phospho-cofilin; RHOQ, Ras homolog family member Q; siRNA, small interfering RNA.

Cofilin is a key downstream effector of CDC42 signaling that regulates actin turnover (1, 32). Active/non-phosphorylated cofilin binds to actin and promotes actin filament disassembly through severing and depolymerization (32, 33). In contrast, inactive (phosphorylated) cofilin loses its actin-binding and severing activities, resulting in the stabilization of actin filaments (34, 35). We previously demonstrated enhanced phospho-cofilin levels in MDCK^Cdc42KD^ cells *via* activated RhoA signaling induced by CDC42-KD (4). First, we confirmed the increased phospho-cofilin levels following transient *Cdc42*-KD using a validated si*Cdc42* (4) in MDCK cells (Fig. 6*A*, >0.5 nM). Second, transient *RhoQ* KD using si*RhoQ-2* also increased phospho-cofilin levels (Fig. 6*B*, >2.5 nM). Finally, combined si*Cdc42* (0.1 nM) and si*RhoQ-2* (1.25 nM) produced a synergistic increase in phospho-cofilin levels in MDCK cells (Fig. 6, *C* and *D*).

## Discussion

CDC42, RHOQ, and RHOJ belong to the same subgroup of Rho-family small GTPases. RHOQ shares approximately 67% aa similarity with CDC42 in humans and mice, and is its closest homolog (36, 37). Although CDC42 has been extensively studied, RHOQ and RHOJ remain relatively understudied. RHOQ is involved in actin nucleation, particularly axon outgrowth (38), and acts synergistically with CDC42 in Toll-like receptor signaling in B-cells (39). Although RHOQ shows functional similarities to CDC42, it also has distinct roles and signaling pathways (40, 41). CDC42 and RHOQ interact differently with the downstream protein collybistin (37). In the present study, although RHOQ alone did not rescue TJ formation in MDCK^Cdc42KD^ cells, *RhoQ*-KD impaired TJ formation. Moreover, additional deletion of *RhoQ* in *Cdc42*-KO mice exacerbated hearing phenotypes in *Cdc42*;*RhoQ*-DKO mice. CDC42 is a known regulator of AJCs (23); however, RHOQ involvement in AJCs has not previously been reported.

We previously reported that HC-specific *Cdc42*-KO mice showed no PCP abnormalities in the cochlea (4), consistent with another report (25). However, *Cdc42*;*RhoQ*-DKO mice exhibited a significantly increased number of fourth-row OHCs. Because PCP abnormalities occur in mice lacking *Cdc42* in both HCs and supporting cells (42), cochlear PCP may depend on CDC42–RHOQ signaling between these cell types. The mild PCP impairment without hair bundle disorientation observed in *Cdc42*;*RhoQ*-DKO mice may result from impaired AJC formation only in HCs. These mice also showed greater OHC loss at P18 than *Cdc42*-KO mice, although OHC loss did not correlate with fourth-row OHC numbers at high frequencies, suggesting that the extra row does not directly cause OHC loss.

The more severe SNHL in *Cdc42*;*RhoQ*-DKO mice compared with *Cdc42*-KO mice, particularly at low frequencies, gradually diminished and disappeared by 6 weeks of age, likely because severely affected mice died earlier. Although the exact mechanism of the earlier deaths is unclear, *Atoh1*, whose promoter was used to delete *Cdc42* and *RhoQ* in our mouse line, is also expressed in the brainstem, especially in respiratory nuclei, and conventional *Atoh1*-KO mice exhibit perinatal lethality (43, 44). Thus, the earlier death of *Cdc42*;*RhoQ*-DKO mice may reflect synergistic roles of CDC42 and RHOQ in essential physiological processes.

Patients with Group I TKS are believed to manifest SNHL (45). All (nine) reported patients with the Y64C mutation (45, 46, 47), both patients with the R66G mutation (8), and all four patients with the R68Q mutation, including our Patient 4, showed SNHL (8, 48). In contrast, patients with Group II (two patients with C81Y/F, one patient with S83P, and one patient with A159V (8, 49)) and Group III (one patient with I21T, two of three patients with Y23C [while one patient manifested SNHL (50)], and all patients with E171K (8, 51)) TKS mutations were described as not having SNHL or SNHL was not mentioned, suggesting no SNHL phenotype. Additionally, the P34Q (52) and E127K mutations are not associated with SNHL (53). Notably, p.E171K affects only isoform 1 (13), leaving the brain-dominant isoform 2 intact and producing milder actinopathies. Thus, SNHL appears characteristic of Group I TKS but uncommon in other CDC42-related disorders.

Our cell-based data demonstrated that Y64C and R68Q are active CDC42 mutants in terms of microvilli and TJ formation. In pulldown assays, Y64C, R66G, and R68Q mutants exhibited a greater tendency to maintain the active conformation than WT, with the R68Q mutant being the most active. The switch II region of CDC42 is critical for interaction with GDIs, GEFs, and GAPs (54). Consistent with the results, the Y64C and R68Q mutants robustly impaired p50GAP-mediated GTP hydrolysis (Y64C are 12-fold more insensitive to GAP than R68Q), whereas the R66G and Y23C mutants mildly impaired p50GAP-mediated GTP hydrolysis (8). Moreover, the Y64C mutant almost completely abolished the response to GEF, ITSN1, whereas the R68Q mutant slightly increased sensitivity (8). Taken together, Y64C and R68Q mutants are constitutively active, but the Y64C mutant may be a milder activating mutant than R68Q, rendering the Y64C mutant to be a more suitable active mutant in HeLa and MDCK cells, although both are harmful during development of the human cochlea. Furthermore, the Y64, R66, and R68 (55, 56) residues (although R68 to a lesser extent (54, 57)) are critical for interactions with RhoGDI. Although the activity of the R66G and Y23C mutants was not as strong as that of the Y64C and R68Q mutants in the present study, the R66G and Y23C mutants lost their binding affinity to various effectors, including formin-like 2 (FMNL2) and IQ motif containing GTPase-activating protein 1 (IQGAP) (R66G) and p21-activated kinase 1 (PAK1), WASP, and FMNL2 (Y23C) (8). Thus, the order of constitutive activity of CDC42 mutants may be influenced and determined by various conditions and timing of their existence and function, such as *in vitro*/*in vivo* settings and types of cell/organ/tissue. Interestingly, all patients with Y64C, R68Q, and Y23C mutations manifesting SNHL (50, 58, 59) simultaneously showed macrothrombocytopenia (MTC); however, MTC is not mentioned in patients with the R66G mutation (8). MTC accompanied by hearing impairment has also been observed in *Cdc42*-KO mice (4, 60), which show activated RhoA signaling with increased phospho-cofilin levels (4); *cofilin*-KO mice (61, 62, 63); patients with DFNA1 (deafness, non-syndromic, autosomal dominant 1) (64) and its model mice (65) caused by constitutively active mutants of DIAPH1 (19, 20, 66), a downstream molecule of RhoA; and *RhoA*-KO mice (67, 68). Together, these observations suggest that impaired regulation of RHOA signaling may lead to both MTC and SNHL.

Mice lacking cofilin and actin-depolymerizing factor (ADF) show abnormal cuticular plates and AJCs in cochlear HCs, leading to an abnormal stereocilia bundle shape and patterning (63). Moreover, imbalanced actin turnover caused by cofilin and ADF activity impedes TJ integrity in intestinal epithelial cells (69, 70). We previously showed that stable *Cdc42*-KD in MDCK cells enhances phospho-cofilin levels through the activated RhoA-ROCK-LIMK pathway, consistent with a report (71), leading to disrupted AJCs in cochlear HCs (4). In the present study, double KD of *Cdc42* and *RhoQ* synergistically enhanced phospho-cofilin levels in MDCK cells, suggesting their combined involvement in actin turnover. Elevated phospho-cofilin may therefore contribute to the more severe hearing phenotype of *Cdc42*;*RhoQ*-DKO mice compared with *Cdc42*-KO mice at least through AJC disruption.

In conclusion, CDC42 and RHOQ function synergistically to establish and maintain cochlear HC integrity. CDC42 mutants that disrupt GTP/GDP cycling likely contribute to SNHL, particularly in Group I TKS. Because patients with the Y64C mutation show craniofacial malformations and brain anomalies (6, 47, 58, 72), including internal auditory canal stenosis and cochlear nerve hypoplasia (see Fig. S4 of Patient 3), and recurrent infections (58, 59, 72), attention is required when diagnosing HL in TKS, because of congenital malformations in the cochlea and cochlear nerve, as well as ear infections (conductive HL). Indeed, Patient 4 in the present study and the patient with the C81Y mutation reported previously both manifested bilateral otitis media (49). Additionally, thyroid hormone is a critical factor for OHC maturation (73); thus, hypothyroidism, a representative symptom of Group I TKS (46, 47, 58, 72), should also be considered during follow-up of SNHL in TKS (Patients 3 (72), and 4). Further studies using longitudinal patient data are required to better understand the relationship between CDC42 functional status and hearing phenotypes in TKS, as well as the natural history of SNHL in TKS associated with hypothyroidism.

## Experimental procedures

### Ethics approval and consent to participate

This study was approved by the Institutional Animal Care and Use Committees of Kobe University (references 26-03-05, 2020-06-04, and 2024-03-03) and conducted in accordance with the Animal Experimentation Regulations of Kobe University. The human study was reviewed and approved by the Institutional Review Board of the University of the Ryukyus (registration number: 20-271-04-00-01) and the ethics committee of Keio University School of Medicine (approval number: 20110262), and performed in accordance with the ethical standards stated in the 1964 Declaration of Helsinki. Written informed consent was obtained from all participants before inclusion in the study.

### Generation of Atoh1-Cre;Cdc42^flox/flox^;RhoQ^flox/flox^ (Cdc42;RhoQ-DKO) mice

We deleted *Cdc42* and *RhoQ* from inner ear HCs of *Atoh1*-*Cre* transgenic mice (74, 75, 76). HC-specific *Cdc42*-KO mice, *Atoh1*-*Cre*^+/−^;*Cdc42*^*flox*/*flox*^ (*Atoh1*-*Cre*^−/−^;*Cdc42*^*flox*/*flox*^ as controls), have been reported previously (4). *RhoQ*^*flox*/+^ mice (39) (RRID:IMSR_JAX:030928) were purchased from Jackson Laboratory (Bar Harbor, ME, USA). *Atoh1*-*Cre*^+/−^;*Cdc42*^*flox*/*flox*^ and *RhoQ*^*flox*/+^ mice were backcrossed to generate *Atoh1*-*Cre*^+/−^;*Cdc42*^*flox*/*flox*^;*RhoQ*^*flox*/+^ and *Atoh1*-*Cre*^−/−^;*Cdc42*^*flox*/*flox*^;*RhoQ*^*flox*/+^ mice and then crossed to obtain *Atoh1*-*Cre*^+/−^;*Cdc42*^*flox*/*flox*^;*RhoQ*^+/+^ (*Cdc42*-KO) and *Atoh1*-*Cre*^+/−^;*Cdc42*^*flox*/*flox*^;*RhoQ*^*flox*/*flox*^ (*Cdc42*;*RhoQ*-DKO). Age- and sex-matched siblings of the *Atoh1*-*Cre*^−/−^ genotype obtained by crossing were used as controls.

Offspring were genotyped *via* polymerase chain reaction (PCR) using the following primer pairs: *Atoh1*-*Cre* (5′-GCATACCTGGAAAATGCTTC-3′ and 5′-CCAGTGAAACAGCATTGCTG-3′; yielding 250-bp product), *Cdc42*^*flox*^ (5′-ATCGGTCACTGTTCTACTTTG-3′ and 5′-TACTGCTATGACTGAAAACCTC-3′; yielding 162-bp product), *RhoQ*^*flox*^ (5′-CAGGGGCCACATTACACCT-3′ and 5′-GTGGGGAGAGAAGCTCACAG-3′; yielding 219-bp product).

Mice were group-housed in the animal facility of Kobe University under specific pathogen-free conditions using an individually ventilated care system (Tecniplast Japan) and were maintained on a 14 h light and 10 h dark cycle at 23 °C ± 2 deg and 50 ± 10% humidity, with food and water provided *ad libitum*. All mice were identified using numbered ear tags. Both male and female mice were used in the analyses unless otherwise indicated (mice < 3 weeks of age were not differentiated based on sex).

### Antibodies

The following antibodies were used: rabbit anti-CDC42 (Cell Signaling Technology [CST], Danvers, MA, USA; Cat# 2462, RRID:AB_2078085); rabbit anti-RHOQ (Proteintech, Cat# 17805-1-AP, RRID:AB_10732837); rabbit anti-phospho-cofilin (Ser3) (77G2) (CST; Cat#3313, RRID:AB_2080597); rabbit anti-ZO-1 (Proteintech; Cat# 21773-1-AP, RRID:AB_10733242); rabbit anti-vinculin (Proteintech, Cat# 26520-1-AP, RRID:AB_2868558); rabbit anti-calnexin (Abcam; Cat# ab22595, RRID:AB_2069006), mouse anti-glyceraldehyde 3-phosphate dehydrogenase (GAPDH) conjugated with HRP and rabbit anti-α-tubulin conjugated with HRP (MBL, Tokyo, Japan; Cat# M171-7, RRID:AB_10699462 and Cat# PM054-7, RRID:AB_10695326, respectively). Validation data for each antibody were obtained from the respective companies.

### Auditory brainstem response (ABR) measurements

Hearing was assessed *via* ABR measurements at the indicated ages in *RhoQ*-KO, *Cdc42*-KO, and *Cdc42*;*RhoQ*-DKO mice and littermate control mice, as described previously (19). Briefly, mice were anesthetized with an intraperitoneal injection of an anesthetic cocktail (medetomidine 0.3 mg/kg, midazolam 4.0 mg/kg, and butorphanol 5.0 mg/kg) and placed on a heating pad to maintain body temperature. BioSigRP Software and TDT System 3 (Tucker-Davis Technologies, Alachua, FL, USA) were used to generate all stimuli and record responses. The ABR waveforms were recorded for 12.8 ms at a sampling rate of 40,000 Hz using a 50 to 5000 Hz passband, and 500 responses were averaged for each sound pressure level (SPL). Clicks and 8–32-kHz tone bursts were presented in 5 dB steps from 90 dB SPL below the threshold to up to 10 dB SPL. Hearing threshold was defined as the lowest sound intensity level at which a recognizable or reproducible wave III was observed. When no response to stimulation at 90 dB was observed, the threshold was set to 100 dB.

### Plasmids

Short hairpin RNA for canine *Cdc42* (nucleotides 197–215 from *ATG* [*sh197*])-resistant human *CDC42* in pEGFP(C1) (Takara Bio, Kusatsu, Japan) has been reported (4). Human *RHOQ* in pEGFP(C1) was prepared by PCR using human *RHOQ* in pCMV-SPORT6 (provided by RIKEN BRC through the National BioResource Project of MEXT, Japan) and an In-Fusion HD cloning kit (Takara Bio) (77). All mutations (Y23C, Y64C, R66G, and R68Q in *CDC42*; Q67L and Y70C in *RHOQ*, corresponding to Q61L, a constitutively active mutant (10), and Y64C in CDC42) were introduced using the QuickChange Lightning Site-Directed Mutagenesis kit (Agilent Technologies, Santa Clara, CA, USA), and named EGFP-CDC42(Y23C), EGFP-CDC42(Y64C), EGFP-CDC42(R66G), EGFP-CDC42(R68Q), EGFP-RHOQ(Q67L), and EGFP-RHOQ(Y70C).

The doxycycline-inducible vector, pRetroX-TetOne-Puro, was purchased from Takara Bio. *sh197*-resistant EGFP-CDC42(WT), EGFP-CDC42(Y23C), EGFP-CDC42(Y64C), EGFP-CDC42(R66G), and EGFP-CDC42(R68Q), and EGFP-RHOQ(WT), EGFP-RHOQ(Q67L), and EGFP-RHOQ(Y70C) were amplified by PCR and cloned into the EcoRI/BamHI sites of the vector using an In-Fusion HD cloning kit. These constructs were named tetEGFP-CDC42(WT), tetEGFP-CDC42(Y23C), tetEGFP-CDC42(Y64C), tetEGFP-CDC42(R66G), tetEGFP-CDC42(R68Q), tetEGFP-RHOQ (WT), tetEGFP-RHOQ (Q67L), and tetEGFP-RHOQ(Y70C).

### Cells

HeLa (19), MDCK (4), and MDCK cells stably expressing *sh197* (targeting *Cdc42*) in *pSUPER*(*neo*) (MDCK^Cdc42KD^) (4) have been previously described. MDCK^Cdc42KD^ cells were cultured with 300 μg/ml G418. To establish MDCK^Cdc42KD^ with stable tetEGFP-CDC42(WT), tetEGFP-CDC42(Y23C), tetEGFP-CDC42(Y64C), tetEGFP-CDC42(R66G), tetEGFP-CDC42(R68Q), tetEGFP-RHOQ(WT), tetEGFP-RHOQ(Q67L), or tetEGFP-RHOQ(Y70C) expression, MDCK^Cdc42KD^ were electroporated using a NEPA21 electroporator (NEPA GENE, Ichikawa, Japan), and then selected using 300 μg/ml G418 and 5 μg/ml puromycin (FUJIFILM). Clone establishment was confirmed by immunoblotting with antibodies against CDC42 and RHOQ. Cells were maintained in D-MEM (FUJIFILM) containing 10% fetal bovine serum (FBS) (Nichirei Biosciences), 100 units/ml penicillin, and 100 μg/ml streptomycin in a 5% CO_2_ humidified incubator at 37 °C.

### Immunoblotting

Cells were lysed by sonication in homogenizing buffer (78) containing a Protease Inhibitor Cocktail (Nacalai Tesque). Total cell lysates were centrifuged at 900*g* for 5 min at 4 °C, and the supernatants were subjected to sodium dodecyl sulfate–polyacrylamide gel electrophoresis (SDS-PAGE) followed by blocking with 1% fat-free bovine serum albumin (BSA) for 1 h at 23 °C and immunoblotting for 2 h at 23 °C using primary antibodies diluted in tris-buffered saline (TBS) containing 0.03% Triton X-100 (TX). Bound primary antibodies were detected with secondary antibody-horseradish peroxidase (HRP) conjugates using an enhanced chemiluminescence detection system (Clarity; Bio-Rad Laboratories) and FUSION SOLO S with Evolution Capt software (Vilber). For quantification, the relative expression levels of phospho-cofilin were normalized to those of vinculin or calnexin.

### Confocal imaging

To analyze the surface preparation of the organ of Corti, anesthetized mice were transcardially fixed with 4% paraformaldehyde in 0.1 M phosphate buffer (pH 7.4) (78). The dissected inner ears were decalcified in 0.12 M ethylenediaminetetraacetic acid (EDTA) for 2 days at 23 °C (19) and then further dissected into apical, middle, and basal turn pieces. Tissues were permeabilized with 0.3% TX, blocked with 5% fat-free BSA, and incubated with Alexa Fluor 488-labeled phalloidin (Thermo Fisher Scientific, Cat# A12381; 1:750) and 4′,6-diamidino-2-phenylindole (DAPI) in TBS containing 0.03% TX for 2 h at 23 °C. The tissues were then rinsed in TBS containing 0.03% TX and mounted on coverslips using ProLong Antifade (Thermo Fisher Scientific).

To examine the rescue effect of CDC42 and RHOQ on TJ formation using MDCK^Cdc42KD^ cells stably expressing doxycycline-inducible plasmids, the cells were incubated with 100 ng/ml doxycycline 8 h after seeding on a 35-mm glass-bottom dish (Mattek, Ashland, MA, USA). Forty-eight hours after incubation, the cells were fixed with 4% paraformaldehyde, permeabilized with 0.3% TX, stained with ZO-1 antibody for 2 h at 23 °C, followed by incubation with Alexa Fluor 594-conjugated secondary antibodies and DAPI for 0.5 h at 23 °C.

Imaging was performed using a confocal microscope (LSM900; Carl Zeiss, Jena, Germany) with a 40× or 63× oil immersion objective. Orthogonal projection images were generated from each z-stack image using ZEN software (Carl Zeiss). The fluorescence intensity profiles across the arrow for green and red channels were analyzed using Zen software and are shown in the graphs. To evaluate ZO-1 staining in MDCK^Cdc42KD^ cells (rescue experiments), the number of cells with ZO-1 staining in more than half of their perimeter was counted, and the percentage (divided by the total cell number identified by DAPI) was calculated. To evaluate ZO-1 staining in WT MDCK cells (KD experiments), the number of cells whose contours were identified by ZO-1 staining was counted, and the percentage (cells with contours of ZO-1 staining) was calculated.

### Pulldown assay for CDC42 activation

Pulldown assay was performed using a Rac1/Cdc42 Activation Magnetic Beads Pulldown Assay kit (Merck, Darmstadt, Germany), according to the manufacturer’s protocol. Briefly, EGFP-CDC42(WT), EGFP-CDC42(Y64C), EGFP-CDC42(R66G), or EGFP-CDC42(R68Q) were electroporated into 2.0 × 10^6^ HeLa cells using a NEPA21 electroporator (NEPA GENE). Forty-eight hours after transfection, the cells were lysed in 400 μl of lysis buffer, and the total cell lysates were centrifuged at 14,000*g* for 5 min at 4 °C. After saving the aliquots (40 μl) for loading control, the residual supernatants were agitated without or with 100 μM GTPγS or 1mM GDP for 30 min at 30 °C. Then, the supernatants were rotated with magnetic beads conjugated with the p21-binding domain (PBD) of PAK1 for 45 min at 4 °C. The precipitates were washed three times using a magnetic rack, and the material absorbed to beads was eluted in Laemmli’s sample buffer. The aliquots of elutants were subjected to SDS-PAGE followed by immunoblotting using a CDC42 antibody. For quantification, the relative amounts of active EGFP-tagged CDC42 proteins were normalized to expression levels of each EGFP-tagged CDC42 protein.

### Transient CDC42 and RHOQ knockdown

The RNAi sequence for canine and human *Cdc42* (siCdc42: 5′-GATTACGACCGCTGAGTTA-3′) has been previously described (4, 79). The siRNAs for canine *RhoQ* (si*RhoQ-1*: 5′-TGCACCAAATGTACCCTTT-3′; si*RhoQ-2*: 5′-CTGTCCATTTCTCTCAAAA-3′) and control were purchased from Nippon Gene. The siRNAs were transfected into MDCK cells using RNAiMAX (Thermo Fisher Scientific), and used in the experiments 48 h later.

### Human study

Genomic DNA was extracted from whole blood using the QIAamp DNA Blood Mini Kit (Qiagen). Whole-exome sequencing was performed using the SureSelect Human All Exon V6 kit (Agilent Technologies) for capture and HiSeq2500 (Illumina, San Diego, CA, USA) for sequencing with 101-bp paired-end reads. The total reads were aligned to GRCh37 using the Burrows-Wheeler Aligner (http://biobwa.sourceforget.net/). Variants were called using GATK Unified Genotyper and ANNOVAR (http://annover.openbioinfomatics.org/en/latest/). Detected variants were validated by Sanger sequencing.

### Quantification and statistical analysis

All data are presented as means ± SD. Between-group comparisons were conducted using an unpaired two-tailed Student’s *t* test, whereas multiple groups were compared using one-way or two-way analysis of variance (ANOVA), followed by Tukey’s or Bonferroni’s *post hoc* tests for pairwise group differences. Statistical analyses were performed using Prism, version 7.0 (GraphPad Software Inc). Differences between groups were considered statistically significant at ∗*p* < 0.05, ∗∗*p* < 0.01, ∗∗∗*p* < 0.001, and ∗∗∗∗*p* < 0.0001.

## Data availability

The data that support the findings of this study are available in the Experimental procedures, Discussion, and Supporting Information of this article.

## Supporting information

This article contains supporting information (4, 79).

## Conflict of interest

The authors declare that they have no conflicts of interest with the contents of this article.
