# Supplementary material for: Hearing loss associated with CDC42 in mice and humans (Takenouchi–Kosaki syndrome): CDC42 and RHOQ synergistically function in cochlear hair cells
Source: J Biol Chem. 2026 Aug 7;302(10):113402. doi: 10.1016/j.jbc.2026.113402 (PMC13559824; doi:10.1016/j.jbc.2026.113402)
Supplement: Supplementary file 1 [file mmc1.docx]

# SUPPORTING INFORMATION

**Hearing loss associated with CDC42 in mice and humans (Takenouchi–Kosaki syndrome): CDC42 and RHOQ synergistically function in cochlear hair cells**

Takehiko Ueyama^1,*^, Akira Ganaha^2,3^, Shunkou Kurasawa^1^, Hiroaki Mohri^1^, Takashi Nakamura^1^, Shigefumi Morioka^1^, Fumiya Kawama^1^, Shota Kitayama^1^, Naoki Oishi^4^, Tadashi Kaname^5^, Keiji Tabuchi^6^, Kenjiro Kosaki^7^, Toshiki Takenouchi^8^, Hirofumi Sakaguchi^1^

List of materials included:

1. Legends for Movies S1–4: Three-dimensional movies, related to Figure 1.

2. Figures S1–S4 and their legends

**1. Supplementary movie legends**

**Movie S1-4: Enhanced phenotypes induced by EGFP-CDC42(Y64C) and EGFP-CDC42(R68Q) mutants.**

EGFP-CDC42(WT), EGFP-CDC42(Y64C), EGFP-CDC42(R66G), and EGFP-CDC42(R68Q) were transfected into HeLa cells. Forty-eight hours after transfection, the cells were fixed and stained with Alexa594-conjugated phalloidin (red) with DAPI (blue). The three-dimensional movies show EGFP-tagged CDC42 for WT (**Movie S1**), Y64C (**Movie S2**), R66G (**Movie S3**), and R68Q (**Movie S4**) proteins, respectively. Cells transfected with EGFP-CDC42(Y64C) or EGFP-CDC42(R68Q) show enhanced phalloidin staining (microvilli) on the apical surface compared with the surrounding non-transfected cells.

Abbreviations: CDC42, cell division cycle 42; EGFP, enhanced green fluorescent protein; WT, wild type; DAPI, 4′,6-diamidino-2-phenylindole.

**2. Supplementary figure and figure legends**

**Fig. S1****: Clinical and genetic findings in Patient 4 with a CDC42 mutation (p.R68Q)**

(**A**) ABR with click stimulation at 3 months of age revealed no detectable response in either ear, based on wave V thresholds between 90 and 105 dB.

(**B**) Reliable pure-tone audiometry data obtained at 4 years of age, showing bilateral severe-to-profound hearing loss. Red circles and blue crosses indicate the hearing levels in the right and left ears, respectively.

(**C**) Axial CT images of the right (a, c) and left (b, d) temporal bones. The normal incudomalleolar joint (white arrows in a and b), stapes superstructure (white arrowheads in c and d), and cochlea (black arrowheads) are shown in both ears (a–d).

(**D**) Sequencing analysis of *CDC42* showing mutation (NM_001791, exon4:c.G203A: p.R68Q). Integrative Genomics Viewer images from next-generation sequencing of *CDC42* (a). Sanger sequencing traces of the patient (b) and parents (c, d) confirm the heterozygous variant. Arrows indicate the variant nucleotide in the patient (a, b) and the corresponding wild-type nucleotides in the mother (c) and father (d).

Abbreviations: ABR, auditory brainstem response; CDC42, cell division cycle 42; CT, computed tomography; IGV, Integrative Genomics Viewer; WT, wild type.

**Fig. S2: Pulldown assay showing that CDC42(Y64C), CDC42(R66G), and CDC42(R68Q) are active mutants**

EGFP-CDC42(WT), EGFP-CDC42(Y64C), EGFP-CDC42(R66G), or EGFP-CDC42(R68Q) were electroporated into HeLa cells. Forty-eight hours after transfection, the supernatants of cell lysates were agitated without or with 100 μM GTPγS or 1mM GDP followed by rotation with magnetic beads conjugated PBD of PAK1. The aliquots of elutants were subjected to SDS-PAGE followed by immunoblotting using a CDC42 antibody. GTPγS was used as positive control.

(**A**) Bands of active EGFP-CDC42 proteins are observed in EGFP-CDC42(WT), EGFP-CDC42(Y64C), EGFP-CDC42(R66G), and EGFP-CDC42(R68Q) even in normal culture conditions (10% FBS) without stimulation (-), and are most significant in EGFP-CDC42(R68Q), followed by EGFP-CDC42(Y64C). Addition of GDP (GDP) eliminates the band in EGFP-CDC42(WT) but not those in EGFP-CDC42(Y64C), EGFP-CDC42(R66G), and EGFP-CDC42(R68Q). The band with GDP is most significant in EGFP-CDC42(R68Q).

(**B**) Active EGFP-CDC42 protein levels were quantified. The symbols in the graph indicate the number of experiments performed (n= 3–4). *P* values by Student’s t-test are shown in the graph. These data suggest that EGFP-CDC42(Y64C), EGFP-CDC42(R66G), and EGFP-CDC42(R68Q) retain the active conformation more than EGFP-CDC42(WT).

Abbreviations: CDC42, cell division cycle 42; EGFP, enhanced green fluorescent protein; GDP, guanosine diphosphate; GTPγS, guanosine 5'-[γ-thio]triphosphate; PBD, p21-binding domain.

**Fig. S****3: Disruption of tight junctions following *RhoQ* knockdown**

(**A**) WT MDCK cells were transfected with control (5 nM), si*Cdc42* (0.5 nM), si*RhoQ-1* (5 nM), or si*RhoQ-2* (5 nM). Forty-eight hours after transfection, cells were fixed and immunostained with an anti-ZO-1 antibody, followed by an Alexa Fluor 594-conjugated secondary antibody and DAPI counterstaining. Scale bars, 20 μm.

(**B**) ZO-1 staining is severely disrupted by CDC42 KD using a validated siCdc42 ^4,79^ and mildly disrupted by RHOQ KD using two independent siRNAs. Quantification is shown (*****P* < 0.0001, one-way ANOVA with Tukey’s post-hoc test; n= 6).

(**C**) RHOQ KD was confirmed by immunoblotting using an anti-RHOQ antibody. Calnexin was used as a loading control. Arrowhead indicates bands of RHOQ. CDC42 KD is shown in Figure 6A.

Abbreviations: CDC42, cell division cycle 42; KD, knockdown; MDCK, Madin–Darby canine kidney; RHOQ, Ras homolog family member Q; siRNA, small interfering RNA; ZO-1, zonula occludens-1; DAPI, 4′,6-diamidino-2-phenylindole.

**Fig. S****4: Temporal bone CT and MR imaging in Patient 3 showing inner ear malformations**

Axial temporal bone CT images of the right (**A**, **C**) and left (**B**, **D**) ears, as well as T2-weighted magnetic resonance (MR) images of the IAC in the right (**E**) and left ears (**F**). The normal malleus (white arrows), incus (black arrows), stapes superstructure (white arrowheads), and cochlea (black arrowheads) are visible in both ears (**A**–**D**). The widths of the cochlear nerve canal (a) and IAC (b) (**A**, **B**). In both ears, the cochlear nerve canal is <1.4 mm, and the maximum width of the IAC is <3 mm, indicating cochlear aperture and IAC stenosis, respectively. Axial MR images demonstrating hypoplasia of the cochlear nerve entering the cochlear modiolus (arrows), with oblique sagittal MR images of the IAC (lower right corner in **E** and lower left corner in **F**) reveals a hypoplastic cochlear nerve (arrows). With IAC stenosis, the cochleovestibular nerves change course from anterior to lateral in the cisternal portion (arrowheads) of both ears.

Abbreviations: CT, computed tomography; IAC, internal auditory canal; MR, magnetic resonance.
